# Normalized Sombor Indices as Complexity Measures of Random Networks

**DOI:** 10.3390/e23080976

**Published:** 2021-07-29

**Authors:** R. Aguilar-Sánchez, J. A. Méndez-Bermúdez, José M. Rodríguez, José M. Sigarreta

**Affiliations:** 1 Facultad de Ciencias Químicas, Benemérita Universidad Autónoma de Puebla, Puebla 72570, Mexico; ras747698@gmail.com; 2 Instituto de Física, Benemérita Universidad Autónoma de Puebla, Apartado Postal J-48, Puebla 72570, Mexico; jmendezb@ifuap.buap.mx; 3 Departamento de Matemáticas, Universidad Carlos III de Madrid, Avenida de la Universidad 30, 28911 Leganés, Madrid, Spain; jomaro@math.uc3m.es; 4 Facultad de Matemáticas, Universidad Autónoma de Guerrero, Carlos E. Adame No. 54 Col. Garita, Acapulco 39650, Mexico

**Keywords:** computational analysis of networks, Sombor indices, degree–based topological indices, random networks

## Abstract

We perform a detailed computational study of the recently introduced Sombor indices on random networks. Specifically, we apply Sombor indices on three models of random networks: Erdös-Rényi networks, random geometric graphs, and bipartite random networks. Within a statistical random matrix theory approach, we show that the average values of Sombor indices, normalized to the order of the network, scale with the average degree. Moreover, we discuss the application of average Sombor indices as complexity measures of random networks and, as a consequence, we show that selected normalized Sombor indices are highly correlated with the Shannon entropy of the eigenvectors of the adjacency matrix.

## 1. Introduction

Given a network G=(V(G),E(G)), the Sombor index of *G*, introduced by I. Gutman in [1], is defined as

(1) $$SO\left(G\right)=\sum_{uv\in E(G)}\sqrt{k_{u}^{2}+k_{v}^{2}},$$

where uv denotes the edge of the network *G* connecting the vertices *u* and *v* and $k_{u}$ is the degree of the vertex *u*. Additionally, the modified Sombor index of *G* was proposed in [2] as

(2) $${}^{m}SO\left(G\right)=\sum_{uv\in E(G)}\frac{1}{\sqrt{k_{u}^{2}+k_{v}^{2}}}.$$

In addition, two other Sombor indices have been introduced: the first Banhatti-Sombor index [3]

(3) $$BSO\left(G\right)=\sum_{uv\in E(G)}\sqrt{\frac{1}{k_{u}^{2}}+\frac{1}{k_{v}^{2}}}$$

and the α-Sombor index [4]

(4) $$SO_{\alpha }\left(G\right)=\sum_{uv\in E(G)}{\left(k_{u}^{\alpha }+k_{v}^{\alpha }\right)}^{1/\alpha },$$

here α∈R. In fact, there is a general index that includes all the Sombor indices listed above: the first (α,β)−KA index of *G* which was introduced in [5] as

(5) $$KA_{\alpha ,\beta }^{1}\left(G\right)=\sum_{uv\in E(G)}{\left(k_{u}^{\alpha }+k_{v}^{\alpha }\right)}^{\beta },$$

with α,β∈R. Please note that $SO\left(G\right)=KA_{2,1/2}^{1}\left(G\right)$, ${}^{m}SO\left(G\right)=KA_{2,-1/2}^{1}\left(G\right)$, $BSO\left(G\right)=KA_{-2,1/2}^{1}\left(G\right)$, and $SO_{\alpha }\left(G\right)=KA_{\alpha ,1/\alpha }^{1}\left(G\right)$. Additionally, we note that $KA_{1,\beta }^{1}\left(G\right)$ equals the general sum-connectivity index [6] $\chi_{\beta }\left(G\right)=\sum_{uv\in E(G)}{\left(k_{u}+k_{v}\right)}^{\beta }.$

Reduced versions of SO(G), ${}^{m}SO\left(G\right)$ and $KA_{\alpha ,\beta }^{1}\left(G\right)$ were also introduced in [1,2,7]. However, when dealing with random networks we use to approximate vertex degrees by average degrees and since average degrees may be less than one, reduced degree-based indices are not amenable for us. Thus, we do not consider reduced Sombor indices here.

Even though Sombor indices were introduced very recently, there are already several works available in the literature where these indices are applied to chemical graphs of interest, see e.g., [4,5,7,8,9,10,11,12,13,14,15,16,17,18]. Additionally, bounds for Sombor indices as well as relations among them and with many other topological indices have been reported in [4,10,19,20,21,22,23,24]. From the application point of view, they have been shown to be useful to to model entropy and enthalpy of vaporization of alkanes [25]. In addition, the Sombor matrix was proposed and studied in [26]. However, to the best of our knowledge, Sombor indices have not been applied to random networks yet; thus in this work we undertake this task.

Here we consider three models of random networks: Erdös-Rényi (ER) networks, random geometric (RG) graphs, and bipartite random (BR) networks. ER networks [27,28,29,30] $G_{ER}\left(n,p\right)$ are formed by *n* vertices connected independently with probability p∈[0,1], while RG graphs [31,32] $G_{RG}\left(n,r\right)$ consist of *n* vertices uniformly and independently distributed on the unit square, where two vertices are connected by an edge if their Euclidean distance is less or equal than the connection radius $r\in [0,\sqrt{2}]$. In addition, we examine BR networks $G_{BR}\left(n_{1},n_{2},p\right)$ composed by two disjoint sets, set 1 and set 2, with $n_{1}$ and $n_{2}$ vertices each such that there are no adjacent vertices within the same set, being $n=n_{1}+n_{2}$ the total number of vertices in the bipartite network. The vertices of the two sets are connected randomly with probability p∈[0,1].

We stress that the computational study of Sombor indices we perform here is justified by the random nature of the network models we want to explore. Since a given parameter set [(n,p), (n,r), or $\left(n_{1},n_{2},p\right)$] represents an infinite-size ensemble of random [ER, RG, or BR] networks, the computation of a Sombor index on a single network is irrelevant. In contrast, the computation of a Sombor index on a large ensemble of random networks, all characterized by the same parameter set, may provide useful *average* information about the full ensemble. This *statistical* approach, well known in random matrix theory studies, is not widespread in studies involving topological indices, mainly because topological indices are not commonly applied to random networks; for very recent exceptions see [33,34].

Therefore, the purpose of this work is threefold. First, we push forward the statistical (computational) analysis of topological indices as a generic tool for studying average properties of random networks; second, we perform for the first time (to our knowledge), a scaling study of Sombor indices on random networks; and third, we discuss the application of selected Sombor indices as complexity measures of random networks.

## 2. Computational Properties of Sombor Indices on Random Networks

### 2.1. Sombor Indices on Erdös-Rényi Networks

In what follows we present the average values of the indices defined in Equations (1)–(5). All averages are computed over ensembles of $10^{7}/n$ ER networks characterized by the parameter pair (n,p).

**Figure 1:**
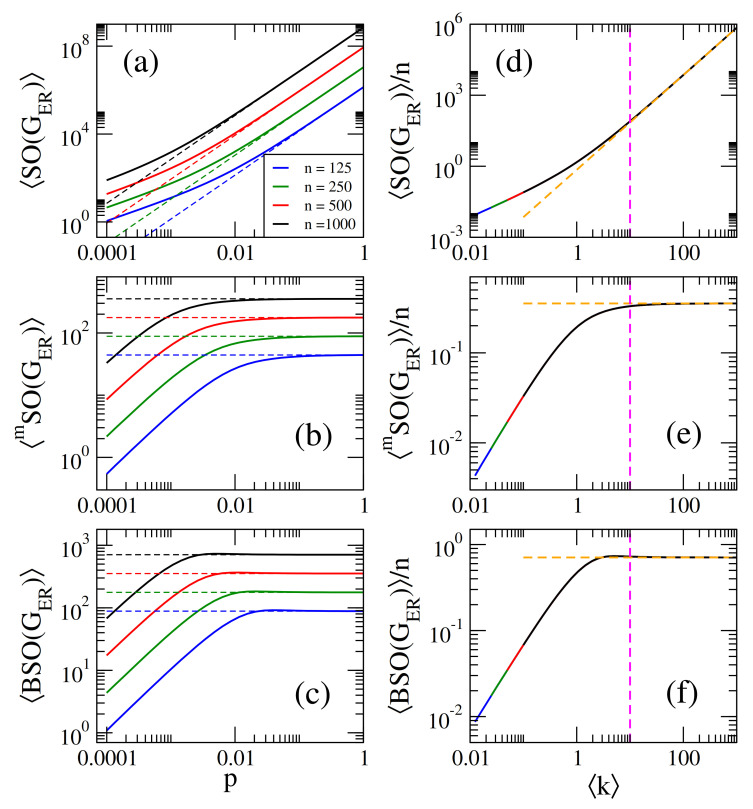
(**a**) Average Sombor index $\langle SO(G_{ER})\rangle$, (**b**) average modified Sombor index $\langle {}^{m}SO\left(G_{ER}\right)\rangle$, and (**c**) average first Banhatti-Sombor index $\langle BSO(G_{ER})\rangle$ as a function of the probability *p* of Erdös-Rényi networks of size *n*. (**d**) $\langle SO(G_{ER})\rangle /n$, (**e**) $\langle {}^{m}SO\left(G_{ER}\right)\rangle /n$, and (**f**) $\langle BSO(G_{ER})\rangle /n$ as a function of $\langle k\rangle$. Dashed lines in panels (**a**–**c**) correspond to Equations (7)–(9), respectively, while dashed lines in panels (**d**–**f**) are Equations (13)–(15), respectively. The vertical magenta dashed line in (**b**–**f**) marks $\langle k\rangle =10$.

**Figure 2:**
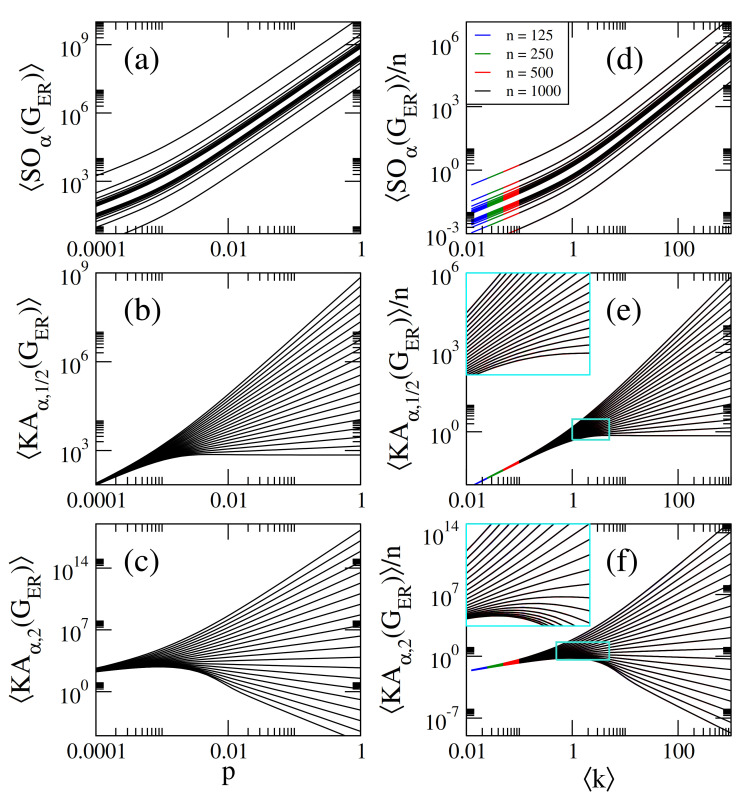
(**a**) Average α-Sombor index $\langle SO_{\alpha }\left(G_{ER}\right)\rangle$, (**b**) average first (α,β)−KA index $\langle KA_{\alpha ,\beta }\left(G_{ER}\right)\rangle$, with β=1/2, and (**c**) average first (α,β)−KA index $\langle KA_{\alpha ,\beta }\left(G_{ER}\right)\rangle$, with β=2, as a function of the probability *p* of Erdös-Rényi networks of size n=1000. In all panels we show curves for α∈[−2,2] in steps of 0.2 (from bottom to top). (**d**) $\langle SO_{\alpha }\left(G_{ER}\right)\rangle /n$, (**e**) $\langle KA_{\alpha ,1/2}\left(G_{ER}\right)\rangle /n$, and (**f**) $\langle KA_{\alpha ,2}\left(G_{ER}\right)\rangle /n$ as a function of $\langle k\rangle$ for ER networks of four different sizes *n*. The insets in (**e**,**f**) are enlargements of the cyan rectangles.

On the one hand, in Figure 1a–c we present, respectively, the average Sombor index $\langle SO(G_{ER})\rangle$, the average modified Sombor index $\langle {}^{m}SO\left(G_{ER}\right)\rangle$, and the average first Banhatti-Sombor index $\langle BSO(G_{ER})\rangle$ as a function of the probability *p* of ER networks of sizes n={125,250,500,1000}. On the other hand, in Figure 2 we plot the average α-Sombor index $\langle SO_{\alpha }\left(G_{ER}\right)\rangle$, see Figure 2a, and the average first (α,β)−KA index $\langle KA_{\alpha ,\beta }^{1}\left(G_{ER}\right)\rangle$, see Figure 2c,d, as a function *p* of ER networks of size n=1000. In Figure 2 we show curves for α∈[−2,2] and, in the case of $\langle KA_{\alpha ,\beta }^{1}\left(G_{ER}\right)\rangle$, we choose to report β=1/2 and β=2 as representative cases.

From this figures we observe that:

(i) The curves of $\langle SO(G_{ER})\rangle$ and $\langle SO_{\alpha }\left(G_{ER}\right)\rangle$ are monotonically increasing functions of *p*. See Figure 1a and Figure 2a.
(ii) The curves of $\langle {}^{m}SO\left(G_{ER}\right)\rangle$ and $\langle BSO(G_{ER})\rangle$ grow for small *p* and saturate above a given value of *p*. See Figure 1b,c.
(iii) The curves of $\langle KA_{\alpha ,\beta }^{1}\left(G_{ER}\right)\rangle$ show three different behaviors as a function of *p* depending on the values of α and β: For $\alpha <\alpha_{0}$, they grow for small *p*, approach a maximum value and then decrease when *p* is further increased. For $\alpha >\alpha_{0}$, they are monotonically increasing functions of *p*. For $\alpha =\alpha_{0}$ the curves saturate above a given value of *p*. For β=1/2 and β=2, the cases reported in Figure 2c,d, we found $\alpha_{0}=-2$ and $\alpha_{0}=-1/2$, respectively.
(iv) When np≫1, we can approximate $k_{u}\approx k_{v}\approx \langle k\rangle$ in Equations (1)–(5), with
  (6) $$\langle k\rangle =\left(n-1\right)p.$$
  Therefore, for np≫1, the average values of the Sombor indices are well approximated by:
  (7) $$\langle SO(G_{ER})\rangle \approx \frac{n}{\sqrt{2}}{\left[(n-1)p\right]}^{2},$$
  (8) $$\langle {}^{m}SO\left(G_{ER}\right)\rangle \approx \frac{n}{2\sqrt{2}},$$
  (9) $$\langle BSO(G_{ER})\rangle \approx \frac{n}{\sqrt{2}},$$
  (10) $$\langle SO_{\alpha }\left(G_{ER}\right)\rangle \approx \frac{n}{2^{1-1/\alpha }}{\left[(n-1)p\right]}^{2},$$
  (11) $$\langle KA_{\alpha ,\beta }^{1}\left(G_{ER}\right)\rangle \approx \frac{n}{2^{1-\beta }}{\left[(n-1)p\right]}^{1+\alpha \beta }.$$
  In Figure 1a–c, we show that Equations (7)–(9) (dashed lines) indeed describe well the data (thick full curves) for large enough *p*. We also verified that Equations (10) and (11) describe well the data for np≫1 reported in Figure 2a–c, however we did not include them to avoid figure saturation.

We note that in Figure 1a–c we present average Sombor indices as a function of the probability *p* of ER networks of four different sizes *n*. It is quite clear from these figures that the curves, characterized by the different network sizes, are very similar but displaced on both axes. A similar observation can be made for $\langle SO_{\alpha }\left(G_{ER}\right)\rangle$ and $\langle KA_{\alpha ,\beta }\left(G_{ER}\right)\rangle$ (not shown in Figure 2a–c to avoid figure saturation). This behavior suggests that the average Sombor indices can be scaled. Then, in what follows we look for the parameters that scale the average Sombor indices.

From Equations (7)–(11) we observe that $\langle X(G_{ER})\rangle \propto nf\left[\left(n-1\right)p\right)]$ or

(12) $$\langle X(G_{ER})\rangle \propto nf\left(\langle k\rangle \right),$$

where *X* and *f* represent all the Sombor indices studied here and the r.h.s. of Equations (7)–(11), respectively. Therefore, in Figure 1d–f and Figure 2d–f we plot average Sombor indices, normalized to *n*, as a function of $\langle k\rangle$ showing that all indices are now properly scaled; i.e., the curves painted in different colors for different network sizes fall on top of each other. Moreover, we can rewrite Equations (7)–(11) as

(13) $$\frac{\langle SO(G_{ER})\rangle }{n}\approx \frac{1}{\sqrt{2}}{\langle k\rangle }^{2},$$

(14) $$\frac{\langle {}^{m}SO\left(G_{ER}\right)\rangle }{n}\approx \frac{1}{2\sqrt{2}},$$

(15) $$\frac{\langle BSO(G_{ER})\rangle }{n}\approx \frac{1}{\sqrt{2}},$$

(16) $$\frac{\langle SO_{\alpha }\left(G_{ER}\right)\rangle }{n}\approx \frac{1}{2^{1-1/\alpha }}{\langle k\rangle }^{2},$$

(17) $$\frac{\langle KA_{\alpha ,\beta }^{1}\left(G_{ER}\right)\rangle }{n}\approx \frac{1}{2^{1-\beta }}{\langle k\rangle }^{1+\alpha \beta }.$$

In Figure 1d–f, we show that Equations (13)–(15) (orange-dashed lines) indeed describe well the data (thick full curves) for $\langle k\rangle \geq 10$. We also verified that Equations (16) and (17) describe well the data for $\langle k\rangle \geq 10$ reported in Figure 2d–f (not shown here to avoid figure saturation).

It is relevant to stress that even when Equation (12) was deduced form Equations (7)–(11), expected to be valid in the dense limit (i.e., for $\langle k\rangle \gg 1$), it is indeed valid for any $\langle k\rangle$ as clearly seen in Figure 1d–f and Figure 2d–f.

### 2.2. Sombor Indices on Random Geometric Graphs

As in the previous Subsection, here we present the average values of the Sombor indices listed in Equations (1)–(5). Again, all averages are computed over ensembles of $10^{7}/n$ random graphs, each ensemble characterized by a fixed parameter pair (n,r).

**Figure 3:**
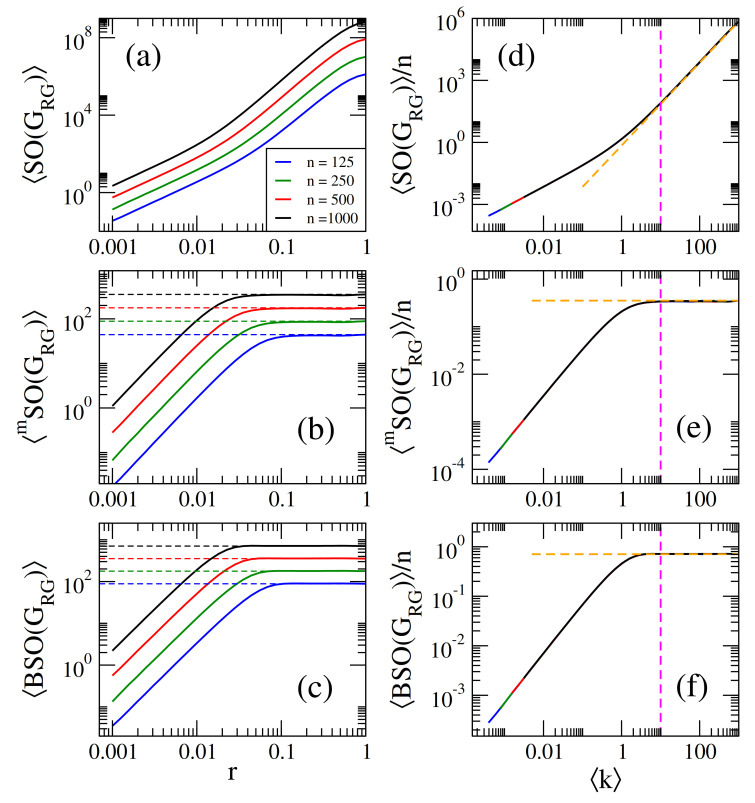
(**a)** Average Sombor index $\langle SO(G_{RG})\rangle$, (**b**) average modified Sombor index $\langle {}^{m}SO\left(G_{RG}\right)\rangle$, and (**c**) average first Banhatti-Sombor index $\langle BSO(G_{RG})\rangle$ as a function of the connection radius *r* of random geometric graphs of size *n*. (**d**) $\langle SO(G_{RG})\rangle /n$, (**e**) $\langle {}^{m}SO\left(G_{RG}\right)\rangle /n$, and (**f**) $\langle BSO(G_{RG})\rangle /n$ as a function of $\langle k\rangle$. Dashed lines in panels (**a**–**c**) correspond to Equations (20)–(22), respectively, while dashed lines in panels (**d**–**f**) are Equations (13)–(15), respectively. The vertical magenta dashed line in (**b**–**f**) marks $\langle k\rangle =10$.

**Figure 4:**
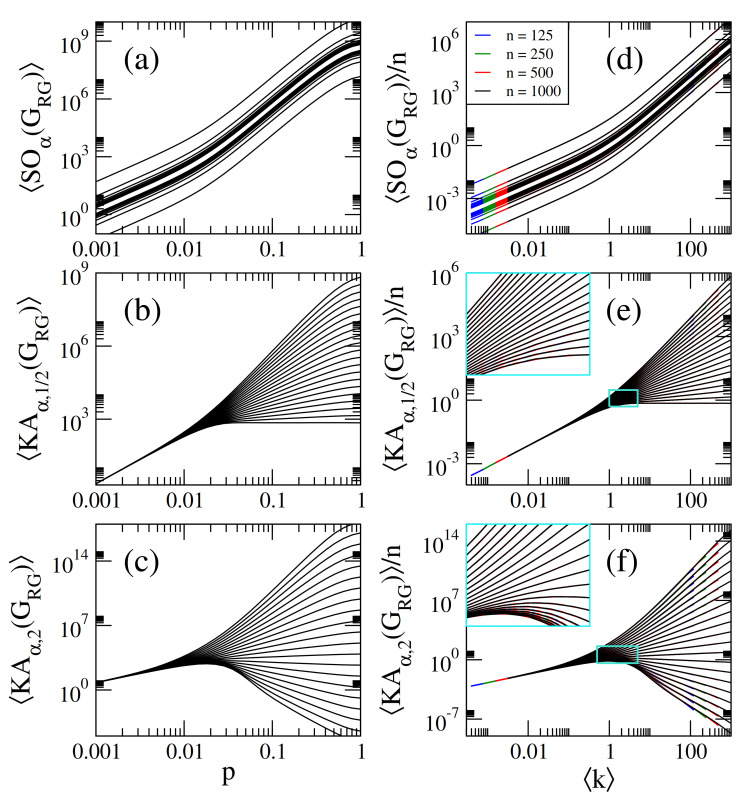
(**a**) Average α-Sombor index $\langle SO_{\alpha }\left(G_{RG}\right)\rangle$, (**b**) average first (α,β)−KA index $\langle KA_{\alpha ,\beta }\left(G_{RG}\right)\rangle$, with β=1/2, and (**c**) average first (α,β)−KA index $\langle KA_{\alpha ,\beta }\left(G_{RG}\right)\rangle$, with β=2, as a function of the connection radius *r* of random geometric graphs of size n=1000. In all panels we show curves for α∈[−2,2] in steps of 0.2 (from bottom to top). (**d**) $\langle SO_{\alpha }\left(G_{RG}\right)\rangle /n$, (**e**) $\langle KA_{\alpha ,1/2}\left(G_{RG}\right)\rangle /n$, and (**f**) $\langle KA_{\alpha ,2}\left(G_{RG}\right)\rangle /n$ as a function of $\langle k\rangle$ for RG graphs of four different sizes *n*. The insets in (**e**,**f**) are enlargements of the cyan rectangles.

Then, in Figure 3a–c we present, respectively, the average Sombor index $\langle SO(G_{RG})\rangle$, the average modified Sombor index $\langle {}^{m}SO\left(G_{RG}\right)\rangle$, and the average first Banhatti-Sombor index $\langle BSO(G_{RG})\rangle$ as a function of the connection radius *r* of RG graphs of sizes n={125,250,500,1000}. Additionally, in Figure 4 we plot the average α-Sombor index $\langle SO_{\alpha }\left(G_{RG}\right)\rangle$, see Figure 4a, and the average first (α,β)−KA index $\langle KA_{\alpha ,\beta }^{1}\left(G_{RG}\right)\rangle$, see Figure 4c,d, as a function *r* of RG graphs of size n=1000.

For comparison purposes, Figure 3 and Figure 4 are similar to Figure 1 and Figure 2. In fact, all the observations **(i–iv)** made in the previous Subsection for ER networks are also valid for RG graphs by replacing $G_{ER}\to G_{RG}$ and p→g(r), with [35]

(18) $$g\left(r\right)=\left\{\begin{array}{cc} r^{2}\left[\pi -\frac{8}{3}r+\frac{1}{2}r^{2}\right] & 0\leq r\leq 1\;,\;\\ \frac{1}{3}-2r^{2}\left[1-\arcsin(1/r)+\arccos(1/r)\right]\; & \;\;\\ \;+\frac{4}{3}\left(2r^{2}+1\right)\sqrt{r^{2}-1}-\frac{1}{2}r^{4} & 1\leq r\leq \sqrt{2}\;. \end{array}\right.$$

However, given the fact that this is the first study (to our knowledge) of average Sombor indices on RG graphs, we want to stress that when nr≫1, we can approximate $k_{u}\approx k_{v}\approx \langle k\rangle$ in Equations (1)–(5), with

(19) $$\langle k\rangle =\left(n-1\right)g\left(r\right).$$

Therefore, in the dense limit, the average values of the Sombor indices on RG graphs are well approximated by:

(20) $$\langle SO(G_{RG})\rangle \approx \frac{n}{\sqrt{2}}{\left[(n-1)g(r)\right]}^{2},$$

(21) $$\langle {}^{m}SO\left(G_{RG}\right)\rangle \approx \frac{n}{2\sqrt{2}},$$

(22) $$\langle BSO(G_{RG})\rangle \approx \frac{n}{\sqrt{2}},$$

(23) $$\langle SO_{\alpha }\left(G_{RG}\right)\rangle \approx \frac{n}{2^{1-1/\alpha }}{\left[(n-1)g(r)\right]}^{2},$$

(24) $$\langle KA_{\alpha ,\beta }^{1}\left(G_{RG}\right)\rangle \approx \frac{n}{2^{1-\beta }}{\left[(n-1)g(r)\right]}^{1+\alpha \beta }.$$

In Figure 3a–c, we show that Equations (20)–(22) (dashed lines) indeed describe well the data (thick full curves) for large enough *r*. We also verified that Equations (23) and (24) describe well the data reported in Figure 4a–c, for large enough *r*, however we did not include them to avoid figure saturation.

It is quite remarkable to note that by substituting the average degree of Equation (19) into Equations (20)–(22) we obtain exactly the same expressions listed in Equations (13)–(17). Therefore, in Figure 3d–f and Figure 4d–f we plot average Sombor indices, on RG graphs, normalized to *n*, as a function of $\langle k\rangle$ showing that all curves are properly scaled. Additionally, in Figure 3d–f, we show that Equations (13)–(15) (orange-dashed lines) indeed describe well the data (thick full curves) for $\langle k\rangle \geq 10$. We also verified (not shown here) that Equations (16) and (17) describe well the data for $\langle k\rangle \geq 10$ reported in Figure 2d–f.

### 2.3. Sombor Indices on Bipartite Random Networks

Now we compute average Sombor indices on ensembles of $10^{7}/n$ BR networks. In contrast to ER and RG networks now the BR network ensembles are characterized by three parameters: $n_{1}$, $n_{2}$, and *p*. Thus, we consider two cases: $n_{1}=n_{2}$ and $n_{1}<n_{2}$. We note that bounds for the Sombor index on bipartite networks have been reported in [21].

**Figure 5:**
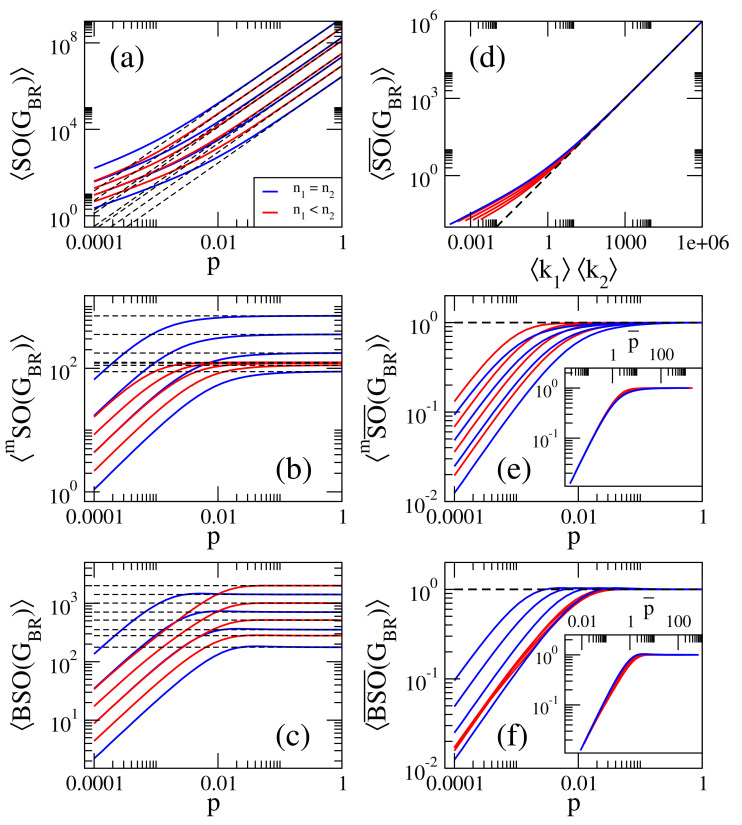
(**a**) Average Sombor index $\langle SO(G_{BR})\rangle$, (**b**) average modified Sombor index $\langle {}^{m}SO\left(G_{BR}\right)\rangle$, and (**c**) average first Banhatti-Sombor index $\langle BSO(G_{BR})\rangle$ as a function of the probability *p* of bipartite random networks with sets of seizes $n_{1}$ and $n_{2}$. In all panels: $n_{1}=n_{2}$ with $n_{2}=\left\{125,250,500,1000\right\}$ (blue lines, $n_{2}$ increases from bottom to top) and $n_{1}<n_{2}$ with $n_{1}=125$ and $n_{2}=\left\{250,500,1000,2000\right\}$ (red lines, $n_{2}$ increases from bottom to top). (**d**) $\langle \bar{SO}\left(G_{BR}\right)\rangle =\langle SO(G_{BR})\rangle /\sqrt{n_{1}^{2}+n_{2}^{2}}$ vs. the product $\langle k_{1}\rangle \langle k_{2}\rangle$. (**e**) $\langle {}^{m}\bar{SO}\left(G_{BR}\right)\rangle =\sqrt{n_{1}^{2}+n_{2}^{2}}\langle {}^{m}SO\left(G_{BR}\right)\rangle /\left(n_{1}n_{2}\right)$ vs. *p*. (**f**) $\langle \bar{BSO}\left(G_{BR}\right)\rangle =\langle BSO(G_{BR})\rangle /\sqrt{n_{1}^{2}+n_{2}^{2}}$ vs. *p*. Dashed lines in panels (**a**–**c**) correspond to Equations (26)–(28), respectively, while dashed lines in panels (**d**–**f**) are Equations (27), (28) and (31) respectively. The inset in (**e**) shows $\langle {}^{m}\bar{SO}\left(G_{BR}\right)\rangle$ vs. $\bar{p}=p\sqrt{n_{1}^{2}+n_{2}^{2}}$. The inset in (**f**) shows $\langle \bar{BSO}\left(G_{BR}\right)\rangle$ vs. $\bar{p}=pn_{1}n_{2}/\sqrt{n_{1}^{2}+n_{2}^{2}}$.

**Figure 6:**
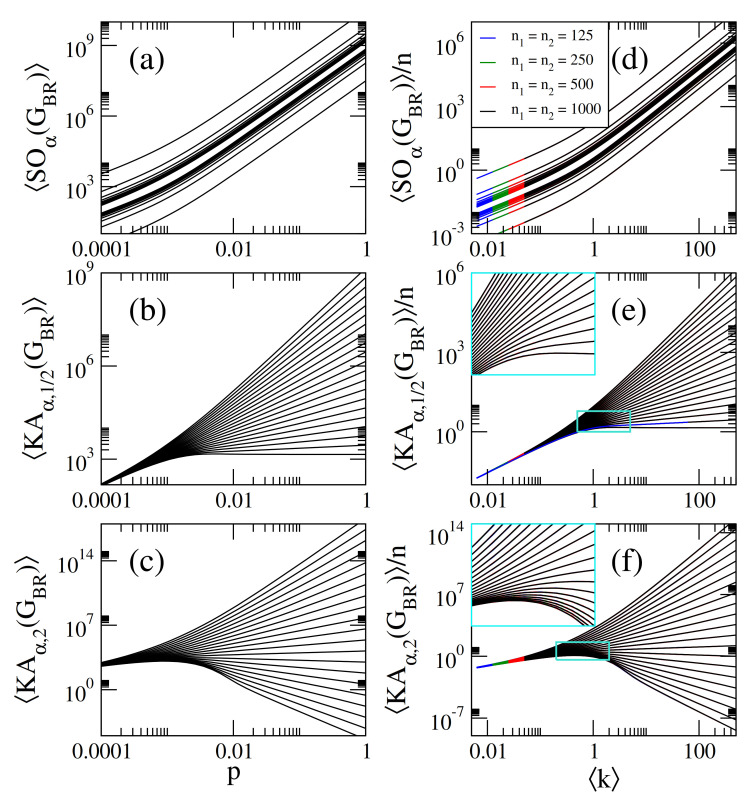
(**a**) Average α-Sombor index $\langle SO_{\alpha }\left(G_{BR}\right)\rangle$, (**b**) average first (α,β)−KA index $\langle KA_{\alpha ,\beta }\left(G_{BR}\right)\rangle$, with β=1/2, and (**c**) average first (α,β)−KA index $\langle KA_{\alpha ,\beta }\left(G_{BR}\right)\rangle$, with β=2, as a function of the probability *p* of bipartite random networks with sets of seizes $n_{1}=n_{2}=1000$. In all panels we show curves for α∈[−2,2] in steps of 0.2 (from bottom to top). (**d**) $\langle SO_{\alpha }\left(G_{BR}\right)\rangle /n$, (**e**) $\langle KA_{\alpha ,1/2}\left(G_{BR}\right)\rangle /n$, and (**f**) $\langle KA_{\alpha ,2}\left(G_{BR}\right)\rangle /n$ as a function of $\langle k\rangle$ for BR networks of four different sizes *n*. The insets in (**e**,**f**) are enlargements of the cyan rectangles.

In Figure 5a–c we present, respectively, the average Sombor index $\langle SO(G_{BR})\rangle$, the average modified Sombor index $\langle {}^{m}SO\left(G_{BR}\right)\rangle$, and the average first Banhatti-Sombor index $\langle BSO(G_{BR})\rangle$ as a function of the probability *p* of BR networks characterized by $n_{1}=n_{2}$ with $n_{2}=\left\{125,250,500,1000\right\}$ (blue lines) and BR networks characterized by $n_{1}<n_{2}$ with $n_{1}=125$ and $n_{2}=\left\{125,250,500,1000\right\}$ (red lines). Additionally, in Figure 6 we plot the average α-Sombor index $\langle SO_{\alpha }\left(G_{BR}\right)\rangle$, see Figure 6a, and the average first (α,β)−KA index $\langle KA_{\alpha ,\beta }^{1}\left(G_{BR}\right)\rangle$, see Figure 6c,d, as a function *p* of BR networks of size $n_{1}=n_{2}=1000$.

It is interesting to notice that all the observations **(i–iv)** made in Section 2.2 for ER networks are also valid for BR networks by just replacing $G_{ER}\to G_{BR}$. Moreover, we can also write approximate expressions for the average Sombor indices on BR networks in the dense limit. However, since edges in a bipartite network join vertices of different sets, and we are labeling here the sets as set 1 and set 2, we replace $d_{u}$ by $d_{1}$ and $d_{v}$ by $d_{2}$ in the expression for the Sombor indices. Thus, when $n_{1}p\gg 1$ and $n_{2}p\gg 1$, we can approximate $k_{u}=k_{1}\approx \langle k_{1}\rangle$ and $k_{v}=k_{2}\approx \langle k_{2}\rangle$ in Equations (1)–(5), with

(25) $$\langle k_{1,2}\rangle =n_{2,1}p.$$

Therefore, in the dense limit, the average values of the Sombor indices on BR networks are well approximated by:

(26) $$\langle SO(G_{BR})\rangle \approx \sqrt{n_{1}^{2}+n_{2}^{2}}{\left(n_{1}n_{2}\right)}^{2}p^{4},$$

(27) $$\langle {}^{m}SO\left(G_{BR}\right)\rangle \approx \frac{n_{1}n_{2}}{\sqrt{n_{1}^{2}+n_{2}^{2}}},$$

(28) $$\langle BSO(G_{BR})\rangle \approx \sqrt{n_{1}^{2}+n_{2}^{2}},$$

(29) $$\langle SO_{\alpha }\left(G_{BR}\right)\rangle \approx {\left(n_{1}^{\alpha }+n_{2}^{\alpha }\right)}^{1/\alpha }{\left(n_{1}n_{2}\right)}^{2}p^{4},$$

(30) $$\langle KA_{\alpha ,\beta }^{1}\left(G_{BR}\right)\rangle \approx n_{1}n_{2}p{\left[{\left(n_{1}p\right)}^{\alpha }+{\left(n_{2}p\right)}^{\alpha }\right]}^{\beta }.$$

Above we used $|E\left(G_{BR}\right)|=n_{1}n_{2}p$. In Figure 5a–c, we show that Equations (26)–(28) (black-dashed lines) indeed describe well the data (thick full curves) for large enough *p*.

As for ER networks, here for BR networks the average modified Sombor index and the average first Banhatti-Sombor index do not depend on the probability *p* in the dense limit, see Equations (27) and (28). Additionally, by recognizing the average degrees $\langle k_{1,2}\rangle$ in Equations (26), (29) and (30), they can be rewritten as

(31) $$\langle SO(G_{BR})\rangle \approx \sqrt{n_{1}^{2}+n_{2}^{2}}{\left(\langle k_{1}\rangle \langle k_{2}\rangle \right)}^{2},$$

(32) $$\langle SO_{\alpha }\left(G_{BR}\right)\rangle \approx {\left(n_{1}^{\alpha }+n_{2}^{\alpha }\right)}^{1/\alpha }{\left(\langle k_{1}\rangle \langle k_{2}\rangle \right)}^{2},$$

(33) $$\langle KA_{\alpha ,\beta }^{1}\left(G_{BR}\right)\rangle \approx \left|E\left(G_{BR}\right)\right|{\left({\langle k_{1}\rangle }^{\alpha }+{\langle k_{2}\rangle }^{\alpha }\right)}^{\beta }.$$

Therefore, by plotting $\langle \bar{SO}\left(G_{BR}\right)\rangle$ vs. $\langle k_{1}\rangle \langle k_{2}\rangle$, $\langle {}^{m}\bar{SO}\left(G_{BR}\right)\rangle$ vs. *p*, and $\langle \bar{BSO}\left(G_{BR}\right)\rangle$ vs. *p* [with $\langle \bar{SO}\left(G_{BR}\right)\rangle =\langle SO(G_{BR})\rangle /\sqrt{n_{1}^{2}+n_{2}^{2}}$, $\langle {}^{m}\bar{SO}\left(G_{BR}\right)\rangle =\sqrt{n_{1}^{2}+n_{2}^{2}}\langle {}^{m}SO\left(G_{BR}\right)\rangle /\left(n_{1}n_{2}\right)$, and $\langle \bar{BSO}\left(G_{BR}\right)\rangle =\langle BSO(G_{BR})\rangle /\sqrt{n_{1}^{2}+n_{2}^{2}}$], see Figure 5d–f, we confirm that the curves of these average Sombor indices on BR networks coincide in the dense limit, as predicted by Equations (27), (28) and (31), respectively.

It is relevant to stress that, while the curves $\langle {}^{m}\bar{SO}\left(G_{BR}\right)\rangle$ vs. *p* and $\langle \bar{BSO}\left(G_{BR}\right)\rangle$ vs. *p* are properly normalized on the vertical axis, they are still not scaled on the *p*-axis, i.e., the curves of the main panels in Figure 5e,f do not coincide. However, through a scaling approach, it is possible to find the scaling parameter $p^{*}=p^{*}\left(n_{1},n_{2}\right)$ such that the curves $\langle {}^{m}\bar{SO}\left(G_{BR}\right)\rangle$ vs. $\bar{p}$ and $\langle \bar{BSO}\left(G_{BR}\right)\rangle$ vs. $\bar{p}$, with $\bar{p}\equiv p/p^{*}$, fall on top of each other. The scaling approach has been successfully used to find universal properties of random graphs and network models, see e.g., [33,34,36,37,38]. We can summarize this approach, as applied to the modified Sombor index and the first Banhatti-Sombor index on BR networks, in the following steps: (i) plot $\langle {}^{m}SO\left(G_{BR}\right)\rangle$ and $\langle BSO(G_{BR})\rangle$ as a function of the parameter *p*, which drives the BR network model from the regime of mostly isolated vertices (MIV) to the regime of mostly connected networks (MCN), so that the MCN regime can be well identified (note that this is done in Figure 5b,c); (ii) normalize $\langle {}^{m}SO\left(G_{BR}\right)\rangle$ and $\langle BSO(G_{BR})\rangle$ such that $\langle {}^{m}\bar{SO}\left(G_{BR}\right)\rangle =1$ and $\langle \bar{BSO}\left(G_{BR}\right)\rangle =1$ in the MCN regime (note that this is done in Figure 5e,f); (iii) define the MIV–to–MCN transition point $p^{*}$ as the value of *p* such that $\langle {}^{m}\bar{SO}\left(G_{BR}\right)\rangle \approx C$ and $\langle \bar{BSO}\left(G_{BR}\right)\rangle \approx C$ with C=0.5 (in fact, any value of C∈(0,1) could be used; however we prefer to define $p^{*}$ at half of the MIV–to–MCN transition where it can be easily extracted); (iv) extract numerically $p^{*}$ from the curves $\langle {}^{m}\bar{SO}\left(G_{BR}\right)\rangle$ vs. *p* and $\langle \bar{BSO}\left(G_{BR}\right)\rangle$ vs. *p*; (v) for several combinations of $n_{1}$ and $n_{2}$, plot $p^{*}$ vs. $\sqrt{n_{1}^{2}+n_{2}^{2}}$ for $\langle {}^{m}\bar{SO}\left(G_{BR}\right)\rangle$ and $p^{*}$ vs. $\sqrt{n_{1}^{2}+n_{2}^{2}}/\left(n_{1}n_{2}\right)$ for $\langle \bar{BSO}\left(G_{BR}\right)\rangle$ (we note that looking at the dependencies $p^{*}$ vs. $\sqrt{n_{1}^{2}+n_{2}^{2}}$ for $\langle {}^{m}\bar{SO}\left(G_{BR}\right)\rangle$ and $p^{*}$ vs. $\sqrt{n_{1}^{2}+n_{2}^{2}}/\left(n_{1}n_{2}\right)$ were indeed educated guesses that we were able to make due to our previous experience on scaling studies, i.e., we used the functions of $n_{1}$ and $n_{2}$ that normalized the average indices $\langle {}^{m}SO\left(G_{BR}\right)\rangle$ and $\langle BSO(G_{BR})\rangle$, respectively. In absence of any hint, one should plot $p^{*}$ vs. $n_{1}$ for fixed $n_{2}$ and $p^{*}$ vs. $n_{2}$ for fixed $n_{1}$ and then deduce the function $p^{*}\left(n_{1},n_{2}\right)$); and (vi) discover the dependencies $p^{*}=p^{*}\left(n_{1},n_{2}\right)$ through power-law fittings. Indeed, we found that $p^{*}=1/\sqrt{n_{1}^{2}+n_{2}^{2}}$ for $\langle {}^{m}\bar{SO}\left(G_{BR}\right)\rangle$ and $p^{*}=\sqrt{n_{1}^{2}+n_{2}^{2}}/\left(n_{1}n_{2}\right)$ for $\langle \bar{BSO}\left(G_{BR}\right)\rangle$. Thus, as can be seen in the instes of Figure 5e,f, the curves of the main panels are now properly scaled when plotted as a function of $\bar{p}$.

It is remarkable to notice that in the case of $n_{1}=n_{2}=n/2$, where $\langle k_{1}\rangle =\langle k_{2}\rangle =\langle k\rangle =np/2$, we obtain exactly the same expressions listed in Equations (13)–(17). This is verified in Figure 6d–f where we plot average Sombor indices on RG graphs, normalized to *n*, as a function of $\langle k\rangle$ showing that all curves are properly scaled.

## 3. General Scaling of Sombor Indices on Random Networks

In the previous Section we have shown that the average value of Sombor indices, normalized to the network size, scale with the average degree $\langle k\rangle$ of the corresponding random network models; we note that this also applies to BR networks when $n_{1}=n_{2}$. This means that $\langle k\rangle$ fixes the average value of any Sombor index for different combinations of network parameters; i.e., the relevant parameter of the random network models we study here is $\langle k\rangle$ and not the specific values of the model parameters. This result highlights the relevance of $\langle k\rangle$ in random network studies. Moreover, the applicability of Equations (13)–(17) to the three random network models we study here allow us to relate the average value of a given Sombor index *X* of the three random network models as

(34) $$\frac{\langle X(G_{ER})\rangle }{n}\approx \frac{\langle X(G_{RG})\rangle }{n}\approx \frac{\langle X(G_{BR})\rangle }{n}\;\;if\;\;\langle k_{ER}\rangle \approx \langle k_{RG}\rangle \approx \langle k_{BR}\rangle ,$$

where $\langle k_{ER}\rangle$, $\langle k_{RG}\rangle$, and $\langle k_{BR}\rangle$ are given in Equations (6), (19) and (25), respectively.

**Figure 7:**
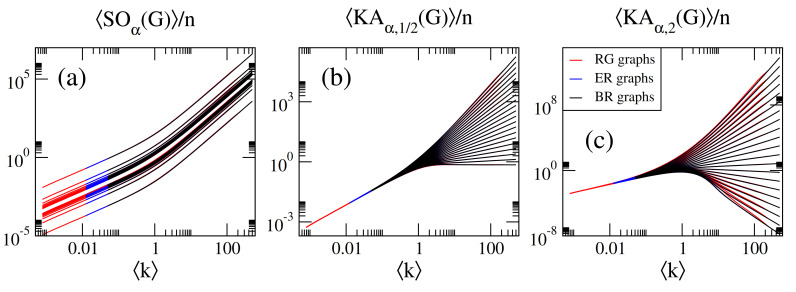
(**a**) $\langle SO_{\alpha }\left(G\right)\rangle /n$, (**b**) $\langle KA_{\alpha ,1/2}\left(G\right)\rangle /n$, and (**c**) $\langle KA_{\alpha ,2}\left(G\right)\rangle /n$ as a function of the average degree $\langle k\rangle$ for RG, ER, and BR networks. In all panels we show curves for α∈[−2,2] in steps of 0.2 (from bottom to top).

Now, to verify Equation (34), in Figure 7 we compare normalized Sombor indices, $\langle X(G)\rangle /n$, for ER, RG, and BR networks, as a function of the corresponding $\langle k\rangle$. Please note that to really put Equation (34) to test, we are using networks of different sizes. Indeed, we observe that Equation (34) is satisfied to a good numerical accuracy; that is, we observe the coincidence of the curves $\langle X(G)\rangle /n$ vs. $\langle k\rangle$ corresponding to different network models.

## 4. Sombor Indices as Complexity Mesures for Random Networks

Additionally, we want to recall that in complex systems research there is a continuous search of measures that could serve as complexity indicators. In particular, random matrix theory (RMT) has provided us with several measures able to distinguish between (i) integrable and chaotic (i.e., non-integrable) and (ii) ordered and disordered quantum systems [39,40]. Such measures are computed from the eigenvalues and eigenvectors of quantum Hamiltonian matrices. Examples of eigenvalue-based measures are the distribution of consecutive eigenvalue spacings, the spectrum rigidity and the ratios between consecutive eigenvalue spacings; while the inverse participation ratios and Shannon entropies are popular eigenvector-based complexity measures [39,40]. It is interesting to notice that all these RMT measures have also been successfully applied to study networks and graphs since they can be computed from the eigenvalues and eigenvectors of adjacency matrices; see e.g., [36,37,41] and the references therein. Therefore, these measures are able to distinguish between networks composed by mostly isolated vertices and mostly connected networks. Additionally, through scaling studies of RMT measures it has been possible to locate the percolation transition point of random network models [36,37]. It is worth mentioning that the scaling study of average Sombor indices performed in this paper has followed a *statistical* RMT approach; that is, from a detailed computational study we have been able to identify the average degree as the universal parameter of our random network models: i.e., the parameter that fixes the average values of the Sombor indices.

Moreover, recently, it was shown for RG graphs that there is a a huge correlation between the average-scaled Shannon entropy (of the adjacency matrix eigenvectors) and two average-scaled topological indices [38]: the Randić index R(G) and the harmonic index H(G). We believe that this is a remarkable result because it validates the use of average topological indices as RMT complexity measures; already suggested in refs. [33,34] for ER random networks. Now, it is important to stress that not every index could be used as a complexity measure. From our experience, we conclude that good candidates should fulfill a particular requirement: they should obtain well defined values in the trivial regimes (just as RMT measures are). For example, a useful complexity measure for random networks should be close to zero in the regime of mostly isolated vertices while it should become constant above the percolation transition. Indeed, this is a property that both $\langle R(G)\rangle$ and $\langle H(G)\rangle$ have: $\langle R(G)\rangle \approx \langle H(G)\rangle \approx 0$ for mostly isolated vertices while $\langle R(G)\rangle /n\approx \langle H(G)\rangle /n\approx 1/2$ once the network is well above the percolation transition.

Therefore, a straightforward application of our study on Sombor indices is the identification of specific Sombor indices as complexity measure candidates. Recall that we particularly require, for an average-scaled Sombor index to work as complexity measure, that $\langle X(G)\rangle /n\approx const.$ for large enough $\langle k\rangle$. In fact, from Equations (14) and (15) we can see that the above condition is fulfilled for $\langle {}^{m}SO\left(G\right)\rangle$ and $\langle BSO(G)\rangle$, respectively. More generally, by properly choosing the values of α and β in Equation (17) we could also use $\langle KA_{\alpha ,\beta }\left(G\right)\rangle$ as complexity measure. Specifically, for β=−1/α we obtain

(35) $$\frac{\langle KA_{\alpha ,-1/\alpha }^{1}\left(G\right)\rangle }{n}\approx \frac{1}{2^{1+1/\alpha }}.$$

**Figure 8:**
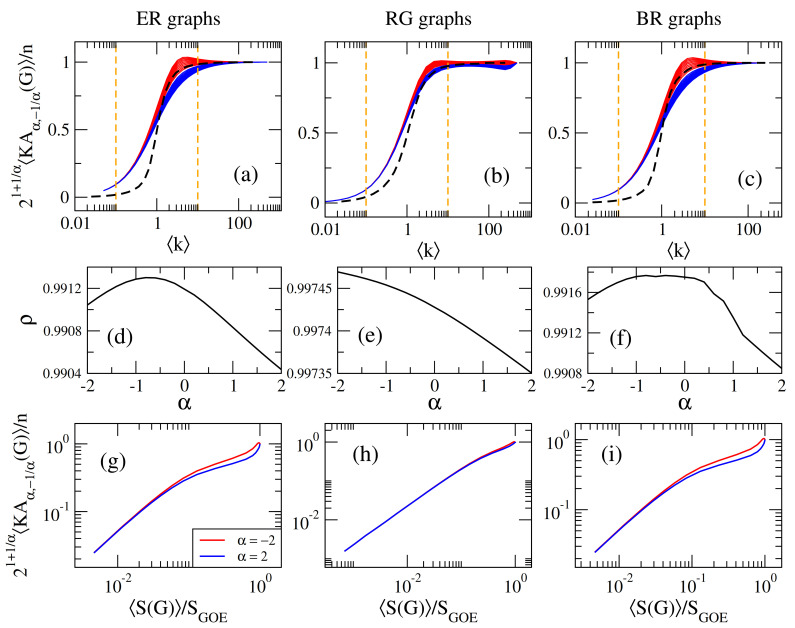
$2^{1+1/\alpha }\langle KA_{\alpha ,-1/\alpha }^{1}\left(G\right)\rangle /n$ as a function of the average degree $\langle k\rangle$ for (**a**) ER networks of size n=500, (**b**) RG graphs of size n=500, and (**c**) BR networks with $n_{1}=n_{2}=250$. In all panels we show curves for α∈[−2,2] in steps of 0.2; except for α=0. Red (blue) lines correspond to α<0 (α>0). Vertical orange dashed-lines mark $\langle k\rangle =1/10$ and $\langle k\rangle =10$, see the text. Black-dashed lines in (**a**–**c**) correspond to the normalized Shannon entropies $\langle S(G)\rangle /S_{\mathrm{GOE}}$. (**d**–**f**) Pearson’s correlation coefficient ρ between $2^{1+1/\alpha }\langle KA_{\alpha ,-1/\alpha }^{1}\left(G\right)\rangle /n$ and $\langle S(G)\rangle /S_{\mathrm{GOE}}$ as a function of α. (**g**–**i**) Scatter plots of $2^{1+1/\alpha }\langle KA_{\alpha ,-1/\alpha }^{1}\left(G\right)\rangle /n$ vs. $\langle S(G)\rangle /S_{\mathrm{GOE}}$ for α=−2 and 2.

Please note that $\langle KA_{\alpha ,-1/\alpha }^{1}\left(G\right)\rangle$ reproduces both $\langle {}^{m}SO\left(G\right)\rangle$ and $\langle BSO(G)\rangle$ when α=2 and α=−2, respectively. Thus, in Figure 8 we plot $2^{1+1/\alpha }\langle KA_{\alpha ,-1/\alpha }^{1}\left(G\right)\rangle /n$ as a function of the average degree $\langle k\rangle$ for ER, RG, and BR networks. From the behavior of the average-scaled indices reported in Figure 8 we can identify three regimes: (i) a regime of mostly isolated vertices when $\langle k\rangle <1/10$, where $2^{1+1/\alpha }\langle KA_{\alpha ,-1/\alpha }^{1}\left(G\right)\rangle /n\approx 0$, (ii) a regime corresponding to mostly connected networks when $\langle k\rangle >10$, where $2^{1+1/\alpha }\langle KA_{\alpha ,-1/\alpha }^{1}\left(G\right)\rangle /n\approx 1$, and (iii) a transition regime in the interval $1/10<\langle k\rangle <10$, which is logarithmically symmetric around the percolation transition point $\langle k\rangle \approx 1$. Accordingly, we propose the use of $\langle KA_{\alpha ,-1/\alpha }^{1}\left(G\right)\rangle$ as complexity measure for random network models.

### Correlation between the Average $KA_{\alpha ,-1/\alpha }^{1}\left(G\right)$ Index and the Average Shannon Entropy

Since we are proposing the use of $\langle KA_{\alpha ,-1/\alpha }^{1}\left(G\right)\rangle$ as a complexity measure for random networks, it is pertinent to compare it to other standard RMT complexity measure. To this end we choose the average Shannon entropy $\langle S\rangle$ of the adjacency matrix eigenvectors.

In particular we construct randomly weighted adjacency matrices, see e.g., [38], such that we obtain well-known RMT ensembles in the limits of: (i) isolated vertices (where we obtain random diagonal adjacency matrices, known in RMT as the Poisson ensemble) and (ii) complete networks (where the adjacency matrices become members of the Gaussian Orthogonal Ensemble (GOE)). Specifically, for the normalized eigenvector $\Psi^{i}$, i.e., $\sum_{j=1}^{n}{\left|\Psi_{j}^{i}\right|}^{2}=1$, *S* is defined as

(36) $$S_{i}=-\sum_{j=1}^{n}{\left|\Psi_{j}^{i}\right|}^{2}\ln{\left|\Psi_{j}^{i}\right|}^{2}\;.$$

Then, we use exact numerical diagonalization to obtain the eigenvectors $\Psi^{i}$ (i=1,…,n) of large ensembles of adjacency matrices and compute $\langle S\rangle$, where the average is taken over all the eigenvectors of all the adjacency matrices of the ensemble.

In Figure 8a–c we present $\langle S(G)\rangle$, normalized to $S_{\mathrm{GOE}}\approx \ln\left(n/2.07\right)$, for ER, RG and BR networks; see the black-dashed lines. From these figures one can observe that $\langle KA_{\alpha ,-1/\alpha }^{1}\left(G\right)\rangle$ and $\langle S(G)\rangle$ are indeed highly correlated. To quantify the correlation, in panels Figure 8d–f we report the corresponding Pearson’s correlation coefficient ρ, which turns out to be approximately equal to one for all the values of α we consider. Finally, to validate the high correlation reported by ρ, in Figure 8g–i we show two examples of scatter plots of $2^{1+1/\alpha }\langle KA_{\alpha ,-1/\alpha }^{1}\left(G\right)\rangle /n$ vs. $\langle S(G)\rangle /S_{\mathrm{GOE}}$.

## 5. Conclusions

In this paper we have performed a thorough computational study of Sombor indices on random networks. As models of random networks we have used Erdös-Rényi networks, random geometric graphs, and bipartite random networks.

Within a statistical random matrix theory (RMT) approach, we show that the average values of Sombor indices, normalized to the order of the network *n*, scale with the network average degree $\langle k\rangle$. Thus, we conclude that $\langle k\rangle$ is the parameter that fixes the average values of Sombor indices on random networks; see Equations (13)–(17) and (31)–(33) and panels (d–f) of Figure 1, Figure 2, Figure 3, Figure 4, Figure 5, Figure 6 and Figure 7. Moreover, it is remarkable that we were able to state a scaling law that includes different network models; see Equation (34) and Figure 7. Please note that Equation (34) underlines the relevance of $\langle k\rangle$ in random network studies; that is, we conclude that $\langle k\rangle$ is the relevant parameter of the network models studied here and not the specific values of the parameters of the models.

Moreover, we discuss the application of Sombor indices as complexity measures of random networks and, as a consequence, we show that the average first (α,β)−KA index (with β=−1/α), normalized to *n*, is highly correlated with the averaged-scaled Shannon entropy of the eigenvectors of the network adjacency matrix; see Equation (35) and Figure 8a–c, i.e., $\langle KA_{\alpha ,-1/\alpha }^{1}\left(G\right)\rangle /n$ may serve as complexity measure for random network models. It is pertinent to mention that the use of Sombor indices as complexity measures for random networks has two advantages as compared with standard RMT measures: First, while computing the Shannon entropy of eigenvectors, due to adjacency matrix diagonalization, requires a cubic time complexity, $O(n^{3})$, computing Sombor indices requires linear time complexity, O(n). Thus, the use of Sombor indices is computationally cheaper than using eigenvalue- or eigenvector-based RMT measures. Second, the Sombor indices we selected as complexity measures, see Equation (35), can be interpreted as a variable descriptor or a general index parametrized by the continuous parameter α∈R. Therefore, in contrast with the standard RMT measures (such as the Shannon entropy), we now have a complexity measure that can be fitted to assess specific network properties; as the variable molecular descriptors do. Indeed, the variability as a function of α of the Sombor indices of Equation (35) can be clearly seen in Figure 8a–c.

We hope that our work may motivate further analytical as well as computational studies of Sombor indices on random networks.

## Data Availability

The data presented in this study are available on request from the corresponding author.
